# Tips and Details for Successful Robotic Myomectomy: Single-Center Experience with the First 125 Cases

**DOI:** 10.3390/jcm11113221

**Published:** 2022-06-05

**Authors:** Lei Dou, Yi Zhang

**Affiliations:** Department of Gynecology, The First Hospital of China Medical University, Shenyang 110002, China; doulei840416@163.com

**Keywords:** robot-assisted surgery, myomectomy, surgical tips and details

## Abstract

With the continuous development of minimally invasive and precise surgical techniques, laparoscopic myomectomy has become a mainstream surgical method due to its aesthetic outcomes and rapid postoperative recovery. However, during laparoscopic myomectomy, clinicians often encounter unfavorable factors, such as limited vision, inaccurate suturing, difficulty in removing tumors, and susceptibility to fatigue in the operating position. In recent years, robot-assisted surgery has been widely used in gynecology. The advantages of this technique, such as a three-dimensional surgical view, reducing the surgeon’s tremor, and the seven degrees of freedom of the robotic arms, compensate for the defects in laparoscopic surgery. The Department of Gynecology in our hospital has accumulated a wealth of experience since robot-assisted surgery was first carried out in 2017. In this article, the surgical skills of the robotic myomectomy process are described in detail.

## 1. Introduction

Uterine leiomyomas (uterine fibroids) are the most common benign tumors of the female reproductive tract. Their etiology is still unclear. Estrogen and progesterone are considered to promote the growth of fibroids. Fibroids are monoclonal tumors with chromosomal abnormalities detectable in 40–50% of karyotypes [1]. The clinical symptoms of uterine fibroids depend on their location in the uterus [2], and include excessive menorrhagia, severe abdominal pain, urinary incontinence, and constipation, and can also lead to infertility, spontaneous abortion, premature delivery, or dystocia. It is very rare for uterine fibroids to develop into malignant leiomyosarcoma [3]. The current treatment strategies for uterine fibroids mainly include three methods: drug treatment [4,5], thermal ablation treatment under ultrasound or radiation guidance [6,7], and surgical treatment [8,9]. The purpose of surgical treatment for uterine fibroids is to remove the fibroids and eliminate clinical symptoms. The choice of surgical technique depends on the age of the patient; the desire to preserve fertility; and factors such as the size, location, clinical symptoms of uterine fibroids, and the surgical skills of the surgeon. Laparoscopic surgery is still the mainstream surgical method for myomectomy, but due to the limitations of laparoscopic technology itself, there are many drawbacks [10]. In 2005, the US Food and Drug Administration (FDA) approved the Da Vinci robotic system for use in gynecological surgery. In recent years, the indications for gynecological robot-assisted surgery have gradually expanded and developed rapidly [11]. How to perfectly combine robot-assisted surgery and myomectomy, maximize the advantages of minimally invasive surgery, and achieve the best surgical results are urgent clinical problems that need to be solved.

## 2. Materials and Methods

### 2.1. Study Design and Population

We selected 125 patients who underwent robotic myomectomy in our gynecology department between November 2017 and January 2022, and selected 110 patients who underwent laparoscopic myomectomy during the same period as a control study. The inclusion criteria were as follows: all patients were confirmed by color Doppler ultrasonography, and malignant transformation was excluded; clinical symptoms such as menorrhagia, abdominal pain, urinary incontinence and constipation, or infertility/miscarriage; single or multiple (larger) fibroids with diameters >5 cm; with or without fertility requirements, but uterus preservation is required; voluntary robotic-assisted surgery or laparoscopic surgery. Exclusion criteria were severe cardiac, liver, kidney, or coagulation disorders, and severe pelvic inflammation. There was no significant difference in general data between the two groups (*p* > 0.05) (Table 1).

### 2.2. Surgical Methods

All patients were under general anesthesia with endotracheal intubation, the bladder lithotomy position was taken, and the urinary catheter was indwelled. Four trocars were punctured in both groups, and the punching position was the same (introduced in Section 3.2), the pneumoperitoneum pressure was maintained at 14 mmHg, and the inhalation velocity was maintained at 10 L/min. Robot-assisted surgery was performed by using the da Vinci Surgical Si™ system (Intuitive Surgical, Sunnyvale, CA, USA). The surgical equipment used for the laparoscopy group was a high-definition 3-D laparoscopy system (Karl Storz, Tuttlingen, Germany).

### 2.3. Evaluation Indicators

The operation time (docking time, suture time, tumor removal time, and total time), number of fibroids removed, intraoperative blood loss, total specimen weight, anal exhaust time, hospital stay, VAS pain scale (12 h after surgery, surgery 24 h after surgery, and 72 h after surgery), symptom improvement, complication, relapse, and total cost (Table 2), and details of hospitalization expenses were evaluated (Table 3).

### 2.4. Statistical Processing

Analyses were conducted using SPSS version 24.0 (IBM Inc., Armonk, NY, USA), and *p*-values < 0.05 were considered statistically significant. Student’s *t*-test was used for continuous variables, and χ2 test was used for categorical variables. The Mann–Whitney U test and Fisher’s exact test were used for non-parametric statistics.

## 3. Surgical Tips and Details 

### 3.1. Preoperative Preparation

First, it is necessary to determine whether the patient has plans to reproduce (the patient’s age, whether she has given birth, comorbidities, and partner’s fertility), has menstrual cramps, and wishes to preserve their uterus. Second, preoperative pelvic ultrasound and pelvic magnetic resonance imaging (MRI) are used to determine the size and location of uterine fibroids and whether other diseases are present. Pelvic ultrasound is the most commonly used method for examining uterine fibroids, which allows patients to be examined conveniently and inexpensively in almost all cases [12]. MRI can provide information on the number and size of fibroids, the relationship with the endometrial cavity and serosal surface, and the boundary with the normal myometrium on the basis of ultrasound examination. However, it should be emphasized that, like ultrasound, MRI cannot diagnose malignant tumors [13]. Particular attention should be paid to patients with increased menstrual flow before surgery, the possibility of endometrial disease should be excluded, and if necessary, the diagnosis should be confirmed by preoperative curettage [14].

### 3.2. Location of the Abdominal Puncture Hole

**Figure 1:**
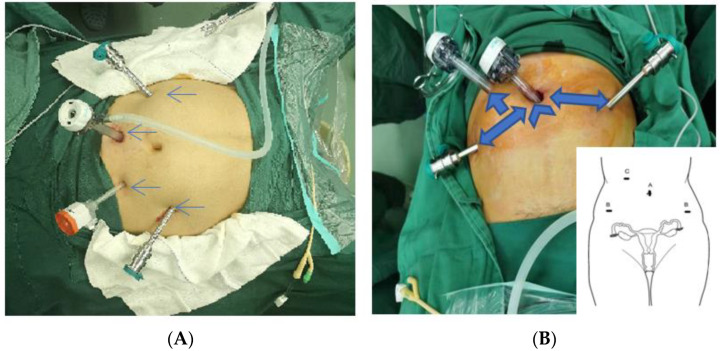
(**A**) The location of the puncture hole in traditional robot-assisted surgery: the camera hole is located at 2–4 cm above the umbilicus (the left side can be opened by 1–2 cm to avoid damage to the retroperitoneal large blood vessels), and the No. 1 arm hole is located at 8–10 cm from the right side of the camera arm, forming an angle of 0–30° with the camera arm (foot side); the No. 2 arm hole is located at 8–10 cm to the left of the camera arm, at an angle of 0–30° with the camera arm (foot side), and the auxiliary hole is located at 5–8 cm above the midpoint of the line connecting the camera hole and No. 1 arm hole. (**B**) Improved puncture hole location for robotic myomectomy: moving the camera hole to the umbilicus follows the principle of Figure 1B, and the puncture positions of other puncture holes follow the principle of Figure 1A.

Since the start of minimally invasive surgery, continuous efforts have been made to select surgical puncture positions and improve puncture safety. However, according to statistics, a large number of the complications of minimally invasive surgery are caused by puncture [15,16]. At present, robot-assisted surgery for benign tumor is performed via abdominal punctures, and the puncture positions are shown in Figure 1A. Based on the experience of robot-assisted surgery in our hospital, we innovatively adopted a 4-hole transumbilical puncture method, as shown in Figure 1B: The first puncture hole (①) is the umbilicus, into which the camera arm is inserted, and the second puncture hole (②) is located at 8–10 cm from the left side of the umbilicus; the No.1 arm is inserted at 0°–30° on the side of the foot; the third puncture hole (③) is 10–12 cm from the right side of the umbilicus; the No.2 arm is inserted at 0°–30° on the side of the foot; the fourth puncture hole (④) is an assistant auxiliary hole, which is located at 5–8 cm vertically above the midpoint of the puncture hole line (① and ②). The advantages of this puncture position design are that the umbilical hole is the thinnest part of the abdominal wall, the puncture path is short, there are fewer blood vessels under the abdominal wall, and subcutaneous emphysema and hematoma are prevented when the tumor is extended. The advantage of surgical tumor removal using the robot avoids the surgical difficulty caused by the collision of instruments in the single-hole robot operation and facilitates the resection and suture of the fibroids [17]. Special attention should be paid to the first puncture hole (①), which is a blind puncture. During puncture, the abdominal wall must be lifted forcefully, and the force and sense of loss must be controlled to avoid damage to the large blood vessels and internal organs. In obese patients or patients with a history of lower abdominal surgery, it is sometimes necessary to enter the open abdomen, and the umbilical area should be strictly disinfected before and during the operation to avoid infection. Depending on the size and location of the fibroids, they should be removed as close as possible to the anterior superior iliac spine and mid-axillary line. The postoperative incision is not easy to detect, and the abdominal wall has good cosmesis. The fourth puncture hole should be located as far away as possible from the other three puncture holes. Depending on the location of the fibroids, one can choose the left or right side under the rib above the midpoint of the two robotic arms to facilitate intraoperative needle delivery and flushing. When all puncture paths are punctured, the trocar is placed perpendicular to the skin and turned 45° downward at the fascia.

### 3.3. Position and Direction of Uterine Incision

**Figure 2:**
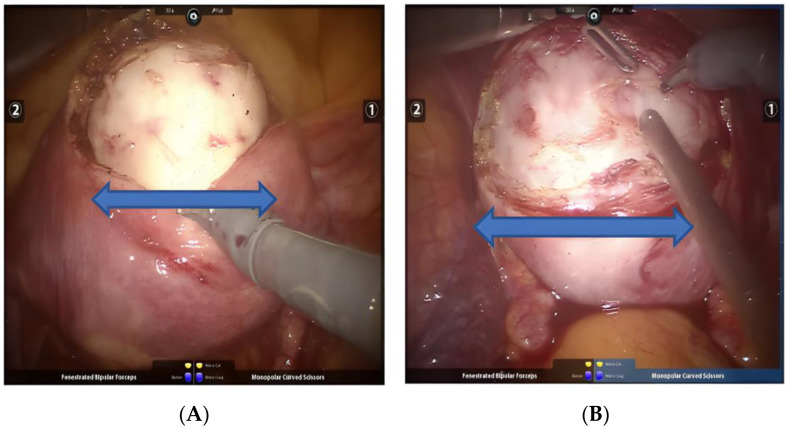
(**A**,**B**) Transverse incision in the anterior (posterior) wall of the uterus. Arrows point to the directions of incision.

At present, there is no consensus on the choice of incision direction during the resection of uterine fibroids. On the basis of preoperative imaging positioning combined with observation under the robot microscope, the most prominent part of the fibroids is often selected, and a unipolar electric shovel or electric scissors are used to make a transverse incision parallel to the direction of the myometrium to reduce damage to the uterus. It is also convenient to extend the incision and suture under the robot microscope [18]; we tend to make only one incision in the front or back, so that multiple fibroids can be removed through one incision (Figure 2A,B). At the same time, the incision should be slightly larger than the largest meridian of the fibroids, which is conducive to the separation and resection of the fibroids. It should be noted that a long incision will not only increase the risk of bleeding and postoperative wound adhesions, but also increase the risk of injury to the fallopian tube opening, bladder, rectum, bilateral parauterine area, and surrounding important organs.

### 3.4. Myomectomy

**Figure 3:**
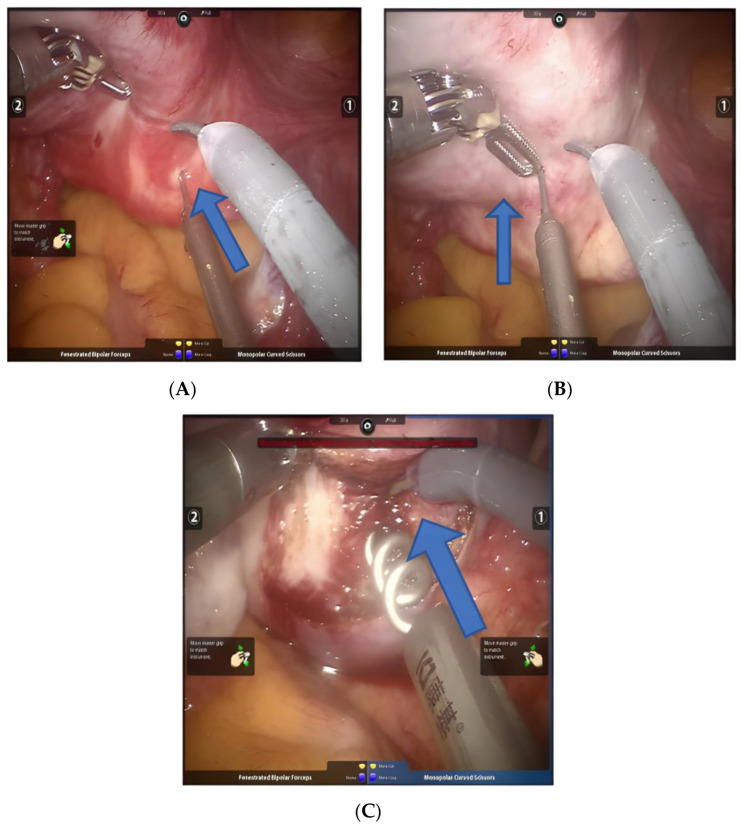
(**A**) Terlipressin was injected into the uterine body. (**B**) “Water cushion method” was used to isolate fibroids. (**C**) The fibroids were pulled with fibroid screws ("Kangji" brand fibroids screws). Arrows point to injection and pulling directions.

A dilute solution of 1 mg of terlipressin +40 mL of normal saline is injected into the normal myometrium of uterine fibroids, and the uterus shrinks after 2–3 min (Figure 3A), which can reduce wound bleeding during the operation. Studies have shown that terlipressin may cause complications, such as heart rate slowing, increased blood pressure, and even cardiac arrest [19]. However, our center has used the above methods and no complications were observed; in contrast, we are concerned about the preoperative heart-related complications. In patients with comorbidities, terlipressin is not used during surgery. Our method of separating fibroids using the “water cushion method” (Figure 3B) is as follows: a 10 mL syringe is connected to the laparoscopic injection needle, the injection needle is inserted around the uterine fibroids from the auxiliary hole, and 50–200 mL of normal saline is injected (when the fibroids are large, the position of the needle can be changed many times if necessary). As the physiological saline forms a “water cushion”, the local color of the uterus changes from pink to white. The serosal layer or muscle layer is cut, and the myoma nodules are bluntly separated and removed, while the pseudocapsule is electrocoagulated to nourish the blood vessels. The advantage of this method is that it is easy to find the level and reduce intraoperative bleeding and residual fibroids. When the fibroids are close to the uterine cavity, the “water cushion” can also be pushed out of the uterine cavity. We use the “water cushion” method to perform robotic resection of uterine fibroids, and no cases of damage to the uterine cavity have been observed. When the fibroids are relatively large, we use fibroid screws (Figure 3C), and constantly change their position, in order to reverse the traction of the fibroids, maintain tension, view the boundary between the fibroids and the endometrium, and move the uterus. The membrane is bluntly peeled from the surface of the fibroids, and the fibroids are then removed from the bed. This can shorten the operation time.

### 3.5. Wound Suture

**Figure 4:**
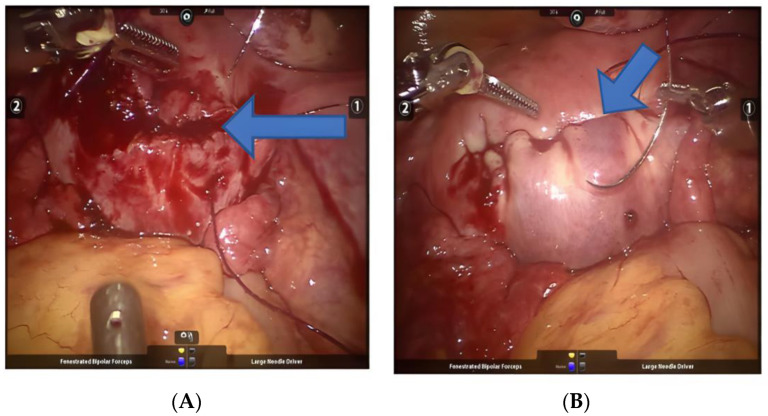
(**A**) The suture of the uterine wound. (**B**) 1-0 V-Loc absorbable barbed suture for continuous suture. Arrows point to suture direction.

The principle of suturing uterine wounds is to reduce dead space, avoid hematoma formation, and provide good reproductive effects for re-pregnancy [20]. Our center adopts a multi-layer continuous suture method [21], using 1-0 V-Loc absorbable barbed sutures to stitch the deep muscle layer of the uterus, the superficial muscle layer of the uterus, and finally the serosal layer of the uterus (Figure 4A,B). For very large fibroids, we suture 5 layers to repair the defective wound. It should be noted that the sutures are often located very deep after the resection of uterine fibroids. Therefore, we prefer to perform 1-0 absorbable thread ligation at the pedicle and remove the fibroid above the ligation thread, so that the bottom can be fully exposed and uterine perforation can be avoided. When stitching all the layers, attention is paid to stitching enough tissue at the same distance to completely eliminate all dead spots for a neat appearance. The advantages of continuous multi-layer suturing with absorbable barbed sutures include a reduction in the difficulty of suturing, shortened operation time, and there is no need for knotting; thus, the entire fibroid bed is exposed without the head, which minimizes the possibility of adhesion formation. Studies have shown that absorbable barbed sutures are firm and reliable, intraoperative bleeding is reduced, and the risk of uterine rupture in subsequent pregnancy is reduced [20]. 

### 3.6. Uterine Fibroid Removal

In current minimally invasive surgery for uterine fibroid removal, due to the different sizes of surgical specimens, most fibroids are larger than the trocar diameter; in particular, large fibroids are more difficult to remove, which is why most clinicians are unwilling to perform minimally invasive uterine fibroid removal. In the past, it was necessary to use a fibroid pulverizer to remove the isolated specimens after rotatory incision; however, because it is difficult in patients with uterine fibroids to determine the nature of the tumor before surgery, residual specimens or malignant tumor tissue dissemination can occur using the fibroid pulverizer during surgery. The risk ultimately affects the patient’s prognosis. In 2014, the US FDA, considering the adverse effects of potential uterine malignancies on the prognosis of patients, issued a notice regarding fibroid pulverizers, and for the first time proposed that fibroid pulverizers are not recommended during laparoscopic surgery [22]. In 2020, the FDA once again updated the shredder safety notice. The FDA continues to recommend limiting the use of laparoscopic morcellation in myomectomy or hysterectomy to ensure that it is used in appropriately specific patients and that only closed morcellation can be performed when a patient is eligible for morcellation [23]. The crushing of uterine fibroids in a closed bag is a remedial option, which can greatly reduce the risk of iatrogenic tissue seeding; however, there are cases of tissue or liquid leakage, and the cost of closing the bag is expensive and difficult to obtain [24]. Second, clinicians also use transvaginal tumor removal and choose the position of the posterior fornix of the vagina. This position is relatively high, and it is difficult to expose the incision and suture. In addition, it is affected by the uterosacral ligaments on both sides of the incision. When the fibroids are large, a larger incision is required. The limitations of this technique include forced traction, which can easily tear or damage the rectum; the transvaginal operation is also restricted by vaginal conditions, which can result in difficult operations, injuries, postoperative infections, postoperative difficulties related to sexual intercourse and impaired fertility [21]. A safe method of removing specimens is currently an issue of concern for gynecologists.

**Figure 5:**
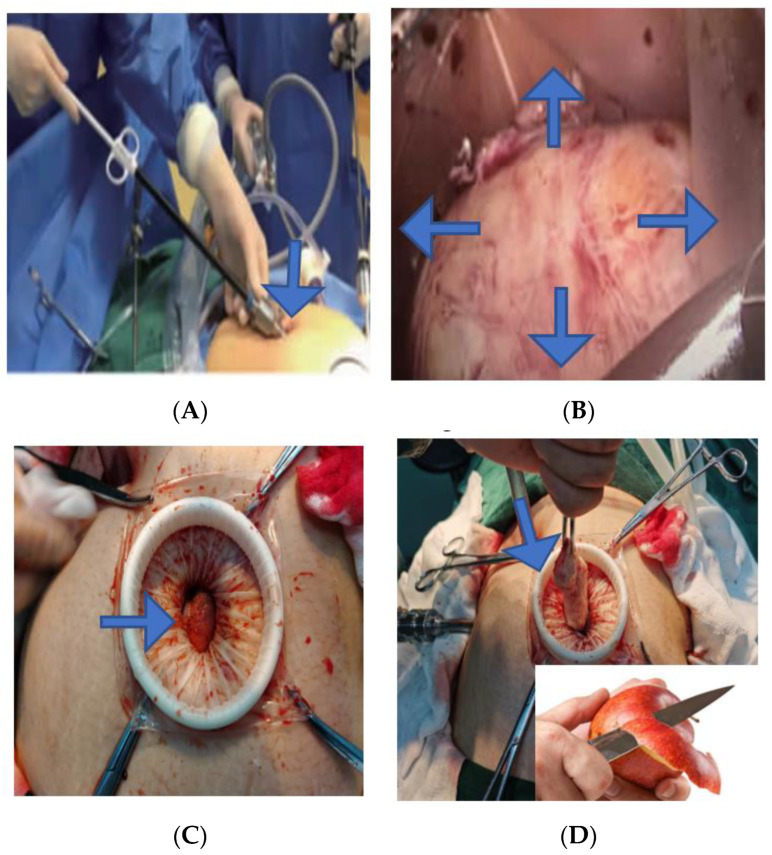

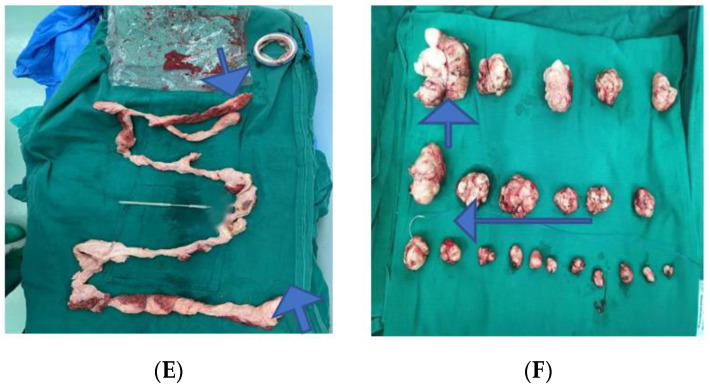
(**A**) The extractor is inserted through an umbilical trocar. (**B**) The fibroids are placed in the specimen bag. (**C**) The incision is dilated with abdominal wall dilator. (**D**) The “peeling” method. (**E**) This is a fibroid removed through the umbilical hole. (**F**) The “bracelet” method. Arrows point to the location and method of fibroids removal and the fibroids removed.

Based on the experience of single-port laparoscopic surgery, our center has used multiple holes to remove tumors through the umbilical hole. By adjusting the position of the puncture in the umbilical hole, the problem of tumor removal in minimally invasive surgery is solved. Thus, this task has become much simpler. This is known as “removing specimens from umbilical isolation”. The specific steps are as follows: 1. Change the position of the camera: change the camera to the second trocar insertion on the right umbilicus and insert the extractor through the umbilicus trocar (Figure 5A). 2. Specimen bagging: unfold the extractor specimen bag and place the fibroids into the specimen bag (Figure 5B). 3. Incision retraction: remove the control lever, slightly expand the umbilical incision, and place the incision retractor in the umbilical area (Figure 5C). 4. Tumor removal: connect the pneumoperitoneum and remove the tumor from the specimen bag under camera monitoring. Through the practice of tumor removal, we have rotated the tumor from the periphery to the center when removing the tumor. Large spherical fibroids are transformed into elongated fibroids and removed through a small incision. It is simple, fast, and easy to learn. We call the tumor removal technique the “peeling” method (Figure 5D,E). This method of tumor removal not only retains the advantages of a multi-hole robotic operation, but also combines the advantages of single-port minimally invasive surgery to remove tumors; thus, robotic myomectomy has the advantages of a tumor-free principle, convenience, and good cosmesis. When the fibroids are smaller, but there are more, we use stitched sutures to place each fibroid into a string, and then pull the sutures to remove the fibroids on a string. We call this the “bracelet” method, which can shorten the operation time and avoid missed specimens (Figure 5F). 

**Figure 6:**
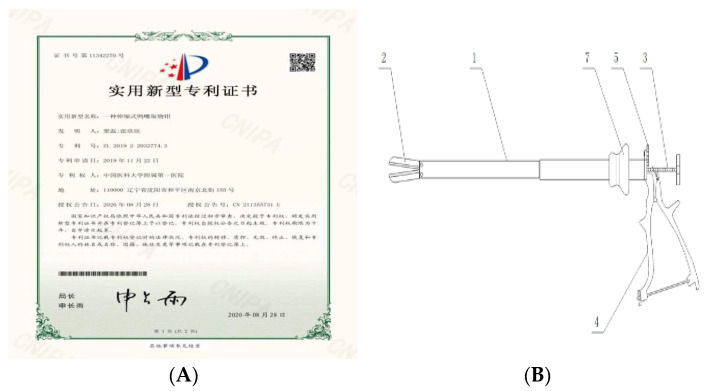
(**A**) The patent for the fibroid extraction forceps. (**B**) The schematic diagram of myoma forceps used for endoscopy. In the picture: 1- telescopic tube, 2- duckbill clamp, 3- pulling device, 4- pressing handle, 5- adjusting gear, 7- rubber locking ring.

We are also studying new tumor removal devices (Figure 6A,B), and hope that in the near future, the tumor removal process can be simplified, so that tumor removal will no longer be the bottleneck in minimally invasive surgery.

### 3.7. Treatment of Several Special Fibroids

**Figure 7:**
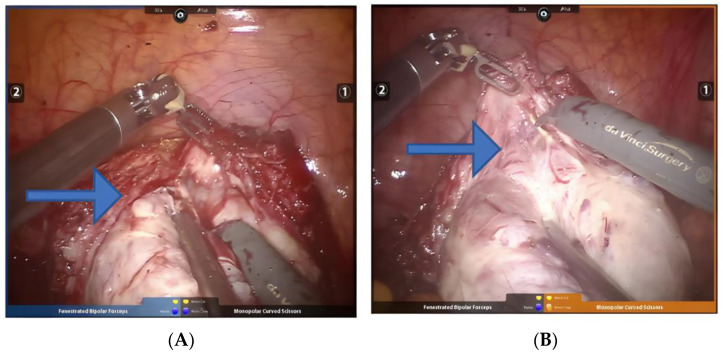
(**A**,**B**) Resection of myomatous with degeneration. Arrows point to the space between the degenerated fibroids and the myometrium.

Fibroids with degeneration: The blood supply of uterine fibroids is provided by the pseudocapsule. The pressure of the pseudocapsule or the tumor pedicle causes the blood supply to the fibroids. Larger fibroids often lack a blood supply in the center, resulting in neoplastic degeneration [25]. Recently, studies have found that pretreatment with drugs such as gonadotropin-releasing hormone agonists is also associated with cystic degeneration [26,27]. It is not easy to identify the boundary during soft resection of uterine fibroids. Errors in levels often lead to residual fibroids or wound bleeding. During the operation, indwelling gauze strips are placed in the surgical area, similar to the method used in ovarian cyst resection, which is separated by robotic arms and 3D vision. The advantage of this ensures the complete resection of degenerative fibroids (Figure 7A,B). It is still necessary to eliminate sarcoma degeneration by rapid pathology during the operation to avoid the occurrence of secondary salvage surgery. Fibroids rapidly increase in size within a short period of time prior to treatment, accompanied by vaginal bleeding. We also assess serum lactate dehydrogenase level, and perform MRI, color flow Doppler and other tests to exclude sarcoma [28,29].

**Figure 8:**
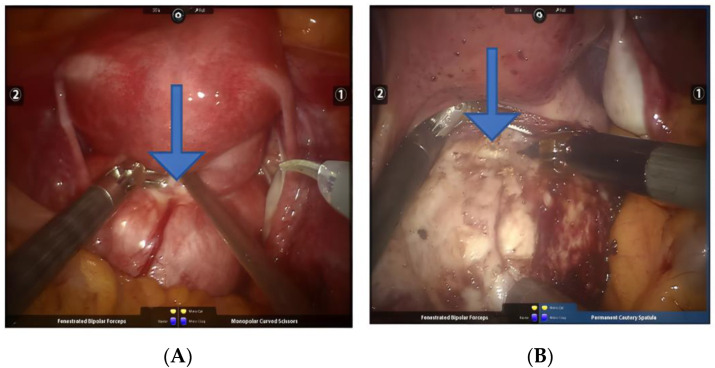
(**A**,**B**) Resection of cervical fibroids. Arrows point to the location of the cervical fibroids.

Cervical fibroids: Cervical fibroids are relatively rare and have little impact on menstruation. Long-term growth can lead to huge cervical fibroids, even beyond the pelvic cavity to the abdominal cavity, with compression symptoms such as abdominal distension and frequent urination [30,31]. As cervical fibroids are closely related to the ureter and rectum, huge cervical fibroids may cause ureter and rectum displacement. During the operation, it is necessary to identify the position and anatomy of the displaced ureter and rectum, and then loosen and free the ureter and rectum. Our center routinely selects preoperative ureteral intubation for this type of operation, which facilitates identification of the ureter during the operation, increases the safety of the operation, and reduces the occurrence of complications. The intraoperative use of uterine lifts is also a reliable way of exposing fibroids. For women who cannot undergo vaginal surgery, suturing can also be performed at the fundus of the uterus. By pulling the sutures, the surgical field can be effectively exposed. We call this the “fishing method”. During the operation, the space between the bladder, cervix and vagina or the space between the uterus and rectum should be separated, and the bladder or rectum should be fully pushed down (Figure 8A,B). At the same time, a transverse incision is made during the operation, and the cervical canal mucosa must be identified when the fibroids are removed to avoid postoperative injury causing cervical canal stenosis and poor menstrual blood flow. At least 2 layers are sutured. Finally, the bladder or rectum covers the wound and restores the anatomical structure.

**Figure 9:**
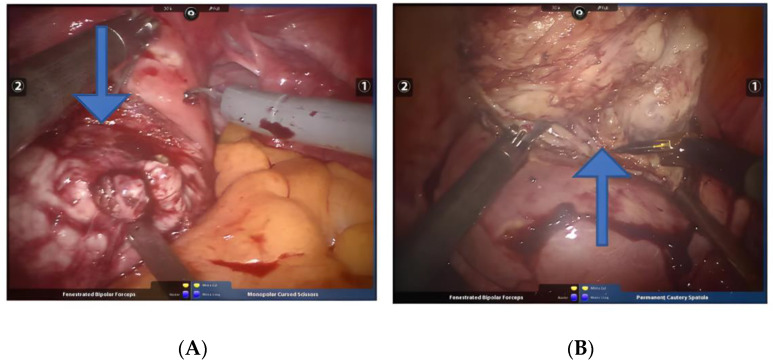
(**A**,**B**) Resection of intravascular leiomyoma. Arrows point to the location of the intravascular leiomyoma.

Intravascular leiomyoma: This is rare in clinical practice. Although it is a benign tumor, it can spread and grow along the vein. There are no specific clinical manifestations in the early stage. When the tumor involves the inferior vena cava, heart, and even the lungs, lower extremity edema, difficult breathing, syncope, pulmonary embolism, and even sudden death can occur [32]. During the operation, the tumor can be found in the parauterine tissue, broad ligament, or uterine isthmus, and a worm-like tumor embolus can be seen in the myometrial vein or parauterine blood vessel, or a bead-like mass can be seen in the dilated pelvic blood vessel, and blood vessels should be monitored for suspected internal leiomyomas, the risk of surgery is often greater [32]. When a fibroid embolus is found in a blood vessel during surgery, robotic bipolar and grasping forceps should be used to peel the fibroids from the blood vessel (Figure 9A,B). The pulling force should be gentle to avoid the rupture of the fibroids. It is difficult to remove the fibroids, and they need to be completely removed along the vein wall to avoid residual fibroids. Whether the tumor is completely removed is closely related to the risk of postoperative recurrence [33].

In recent years, minimally invasive surgery for myomectomy has become a mainstream technique due to good cosmesis of the laparoscopic minimally invasive incision and rapid postoperative recovery, and it is now favored by an increasing number of doctors and patients. However, some scholars have criticized the deficiencies of laparoscopic myomectomy, such as insufficient sutures, improper hemostasis, and an increased chance of uterine rupture in subsequent pregnancy [34]. Although current data show that robotic myomectomy has the advantages of less intraoperative bleeding, less postoperative pain, shorter hospital stay, and fewer complications [35,36], it is expensive, with high maintenance costs, postoperative recurrences, and fertility issues. There is no obvious advantage in function, and this is an unavoidable problem [37]. With the popularization and development of robot-assisted surgery in gynecology, we will also conduct randomized controlled trials via laparoscopic, robotic, vaginal and laparotomy myomectomy techniques, including cost evaluation, calculation of hospital stay, postoperative complications, return to normal life, and rehabilitation to comprehensively decide whether robot-assisted surgery should be the first choice for gynecological myomectomy, or a choice of gynecological surgical technique for clinicians.

### 3.8. Postoperative Care and ERAS Management

Robotic myomectomy has little interference with the abdominal cavity of the patients, and their general condition is good after surgery. In this study, the whole process of postoperative nursing was combined with the concept of ERAS. Within 6 h after the operation, the vital signs were closely observed, the patient was in a low semi-recumbent position, and the patient was assisted to turn over once every two hours.The patient was instructed to breathe regularly, soft music was played in the ward, the patient’s attention was diverted, the patient’s “VAS” score dynamically assessed and accurately recorded, and multimodal analgesia was implemented for the patient; 12 h after the operation, psychological counseling, removal of the urinary catheter, and encouragement of the patient to leave the hospital were conducted. Bed activity and spontaneous urination can prevent lower extremity venous thrombosis; 24 h after surgery, sham feeding (chewing gum) and early eating can protect intestinal mucosal function, prevent dysbacteriosis and ectopic, promote intestinal function recovery, and reduce perioperative complications. Symptoms: 48 h after surgery, incision dressing change, discharge guidance, including prohibiting sexual activity for 2 months, strengthening nutrition, eating a light and easily digestible diet rich in nutrients, weekly follow-up by telephone from 2 months after surgery. Outpatient review: Careful postoperative care is the key to ensuring the smooth recovery of robot-assisted surgery patients after surgery, and also greatly increases patient satisfaction with medical treatment.

## 4. Discussion

Surgery is an important means of treating uterine fibroids. The main goal of myomectomy is to remove the fibroids to relieve symptoms and rebuild the uterus, maintaining the integrity of the uterus, thereby also providing patients with options for future pregnancy and childbirth. Laparoscopy is the most common surgical modality used by gynecologists [10]. Most scholars believe that patients with fibroids larger than 10 cm in diameter, more than 4 in number, and close to submucosal fibroids, as well as cervical fibroids, are relative contraindications to laparoscopic surgery, causing difficulty and even complications [38,39,40]. Robot-assisted systems have their own unique advantages. First, the three-dimensional imaging of the robot-assisted system and the magnification of 10 to 15 times enable the operator to have a clearer, three-dimensional field of view; second, the instruments equipped with the robot-assisted system have a range of motion of 7 degrees of freedom, making it easier to operate in the abdominal cavity; third, the robot system can filter hand tremors, making the operation more stable and safe; finally, the operator operates the robot system in a sitting position, which greatly reduces the operator’s physical exertion, and the operator can operate in a relatively comfortable posture, reducing the occurrence of intraoperative errors [41]. Advincula et al. [42] first described robotic myomectomy in 2004, and many subsequent studies have also shown that robot-assisted surgery has excellent maneuverability, which has brought great changes to myomectomy and expanded minimally invasive surgery. Compared with the laparoscopic group, the robotic group had less blood loss, fewer complications, and a shorter anal exhaust time [43,44]. The length of hospital stay was short, consistent with literature reports [45,46,47]. However, the operation time was short (*p* < 0.05), which is different from the literature [47,48]. The reason for this is that the stable treatment team and mature operation experience shortened the time of tedious preparation (docking robot and connecting instruments) before operation, and the average connection time was approximately 8.8 min; at the same time, the operator had certain experience in laparoscopic operation, combined with the advantages of the three-dimensional vision of the robot and the multi-directional rotation of the instrument, so that the incision, hemostasis, suturing, and other operations were more accurate, and the operation time was significantly shortened. In the study, we also found that the robot-assisted surgery also has advantages for the removal of uterine fibroids in special parts. Under the three-dimensional imaging and magnification effect of the robot-assisted system, the surrounding organs, such as the uterine artery, bladder, ureter, and rectum, can be more clearly exposed, and the fibroids can be more accurately distinguished and removed from the surrounding organs. The wrist of the robotic instrument can be rotated in multiple directions to reach the position that the laparoscopic instrument cannot reach, which is more conducive to precise resection, reduces bleeding, shortens the suturing time, and reduces surgical complications. At present, there are few studies on robots in special parts of uterine fibroids [49], and robots may challenge more complex surgeries in the future.

Of course, the robot-assisted system also has its drawbacks. The surgical cost of the robot-assisted surgery is relatively high, which is not plausible for all patients. In this study, we can see that robotic myomectomy is approximately twice more expensive compared to laparoscopic myomectomy. The main driver of the increase in cost is the fixed cost of the robot. After excluding the surgical fee of robot-assisted surgery, the other total costs of the robot group and the laparoscopic group are comparable or even lower than the laparoscopic group (Table 3). The main reason for the difference in the total hospitalization costs between the robotic group and the laparoscopic group is the difference in operating costs, which to a certain extent reflects the technical value, including less intraoperative blood loss, shorter hospital stay, fewer complications, and rapid postoperative recovery. These advantages also save costs for ward staff and other interventions, which can offset some of the additional costs [50,51]. Some studies have shown that even surgeons without laparoscopic operation experience can master robot-assisted surgery techniques [52]. Compared with laparoscopy, robot-assisted surgery is easier to operate, can significantly reduce the difficulty of surgery, shorten the learning curve, and greatly reduce the operating pressure and burden of the operator [53]. These advantages cannot be simply explained by statistics. Under the current cost distribution model, increasing the volume of robotic surgery will compensate for this disadvantage to a certain extent. As a result, more patients will undergo robot-assisted surgery each year, or if the purchase price of the robot-assisted system is reduced, robot-assisted surgery will be more cost-effective [54]. At the same time, robot-assisted surgery and laparoscopy also have the defect of poor tactility, so they cannot completely replace laparotomy. Laparotomy myomectomy is suitable for large fibroids that are difficult to solve using a robot and multiple uterine fibroids that cannot be completely removed [55,56]. The experience of the surgeon and the size of the center are also factors in the selection of the surgical plan. For clinicians, the robotic surgery system has the characteristics of simple operation, short learning cycle, and rapid growth. With the accumulation of experience of the surgeon, the manipulation of the robotic surgical system has become more proficient, and clinicians are more inclined to perform myomectomy through robot-assisted surgery with a three-dimensional field of view and a stable and flexible operation. Of course, in the choice of surgical methods, both the objective condition and the patient’s economic conditions must be respected. For patients with average economic conditions, laparoscopic myomectomy can also achieve good therapeutic effects. Large-scale medical centers are located in economically developed cities. Patients who seek medical treatment have better economic conditions and higher surgical expectations. The robotic surgery system can meet the needs of patients to the greatest extent, and the development of robotic surgery is also in a leading position. Robotic surgical systems have developed rapidly in the past 10 years, broadening the indications to cover almost all complex gynecological diseases, such as deep invasive endometriosis [57,58], retroperitoneal tumors [59,60], uterine prolapse [61], and uterine transplant [62]. With the development of science and technology, robot technology will also continue to innovate and develop. In the future, the robotic surgical system will be improved and perfected towards being more intelligent, small, and reasonably priced. The introduction of tactile gloves [63], cell image navigation [64], fluorescence imaging [65], and other technologies into robotic surgery can help doctors determine key anatomical structures, determine tumor boundaries in solid organs, and evaluate blood perfusion in target tissues. The surgical instrument can be advanced to the surgical site through a non-linear path, thereby increasing the flexibility of the operation and breaking through the limitations of the surgical scope [66]. Just as the current laparoscopic technology is gradually replacing the traditional open surgery, with the rapid development of science and technology in the medical field, robot-assisted surgery will eventually give full play to its advantages, eradicate constraints, and be widely used in the field of gynecology. Therefore, the advantages of robot-assisted surgery still need to be investigated more deeply over a longer period of time.

This study also has certain shortcomings. A single-center retrospective study has a small number of study samples and lacks the evaluation of long-term efficacy (pregnancy outcome). A study with a larger sample and longer follow-up time is necessary.

## 5. Conclusions

Robot-assisted surgery is safe and feasible for myomectomy, and it has the advantages of more accurate incision, hemostasis, and suturing, as well as a shorter operation time than laparoscopic surgery. During the operation, surgical techniques and methods, such as single incision, “water cushion” separation, the layered suture of barbed lines, the “stripping” method, and the “bracelet” method, can be used to remove tumors in order to reduce the difficulty of surgery. Robot-assisted surgery is also suitable for difficult and specialized myomectomy. Postoperative care and ERAS management can facilitate the recovery of robotic myomectomy patients. We can envision that with the development of robotic-assisted surgical systems and the continuous exploration of surgical techniques by clinicians, this technology is expected to lead the way in gynecological surgery in the future.

## Figures and Tables

**Table 1:** Demographic characteristics.

	Robot (*n* = 125)	Laparoscope (*n* = 110)	*p* Value
Median age (mean, range) (y)	35 (36.6, 23–48)	38 (37.1, 24–52)	0.59
Median BMI (mean, range)	26 (26.7, 18–33)	24 (25.1, 20–31)	0.61
Preoperative symptoms			
Increased menstrual flow	68 (54%)	64 (58%)	0.56
Urinary incontinence/constipation	29 (23%)	23 (21%)	0.67
Abdominal pain	19 (15%)	16 (15%)	0.89
Infertility/miscarriage	9 (7.2%)	7 (6.4%)	0.80
Fibroids			
Single	49 (39%)	39 (35%)	0.55
Multiple	76 (61%)	71 (65%)	
Maximum diameter (Mean, range) (cm)	7.9 (7.3, 5–13)	7.1 (7.0, 5–12)	0.83
Fibroid degeneration	28 (22%)	21 (19%)	0.53
Cervical fibroids	11 (8.8%)	7 (6.4%)	0.48
Broad ligament fibroids (including endovascular leiomyomatosis)	12 (9.6%)	7 (6.4%)	0.36
Intraperitoneal disseminated leiomyomatosis	2 (1.6%)	1 (0.9%)	0.64
History of pelvic surgery	53 (42%)	44 (40%)	0.71

Table 1 summarizes the patient characteristics of the robotic and laparoscopic groups in terms of age (median 35 vs. 38, *p* = 0.59); BMI (median 26 vs. 24, *p* = 0.61); preoperative symptoms, including increased menstrual flow (68 vs. 64, *p* = 0.56), urinary incontinence and constipation (29 vs. 23, *p* = 0.67), abdominal pain (19 vs. 16, *p* = 0.89), and infertility/miscarriage (9 vs. 7, *p* = 0.80); number of fibroids (49 vs. 39 for single, 76 vs. 71 for multiple, *p* = 0.55); largest diameter (median 7.9 vs. 7.1, *p* = 0.83); fibroids in special locations, including fibroid degeneration (28 vs. 21, *p* = 0.53)), cervical fibroids (11 vs. 7, *p* = 0.48), broad ligament fibroids (including intravascular leiomyomatosis) (12 vs. 7, *p* = 0.36), and intraperitoneal disseminated leiomyomatosis (2 vs. 1, *p* = 0.64). There was no significant difference in the history of pelvic surgery (53 vs. 44, *p* = 0.71).

**Table 2:** Intraoperative and postoperative conditions.

	Robot (*n* = 125)	Laparoscope (*n* = 110)	*p* Value
Operation time (min)			
Docking time	8.8 (6.1–15.4)	0	0.00 **
Suture time	22 (14–35)	41 (21–59)	0.00 **
Tumor retrieval time	6 (4–15)	5 (3–21)	0.59
Total time	72 (46–105)	96 (72–135)	0.01 **
Number of fibroids removed	4.8 (1–9)	4.4 (1–7)	0.50
Median blood loss (mL)	45 (5–200)	75 (10–300)	0.01 **
Total specimen weight (g)	420 (180–780)	400 (150–695)	0.66
Anal exhaust time (h)	12 (3–24)	18 (6–39)	0.00 **
Hospital stay (d)	2 (1–5)	3 (1–6)	0.02 *
Pain scale (VAS)			
12 h after surgery	4.2 (2–5)	4.1 (2–6)	0.34
24 h after surgery	3.1 (2–4)	3.0 (2–5)	0.49
72 h after surgery	1.3 (0–3)	1.4 (0–4)	0.55
Symptom improvement	113 (90%)	103 (94%)	0.36
Complications	3 (2.4%)	12 (11%)	0.01**
Relapse	9 (7.2%)	11 (10%)	0.44
Total cost (RMB)	51,231 (47,114–63,587)	26,899 (24,503–30,218)	0.00 **

Table 2 summarizes the findings that the intraoperative and postoperative conditions, the time of tumor removal (6 vs. 5, *p* = 0.59), the number of myomectomies (4.8 vs. 4.4, *p* = 0.50), the total specimen weight (420 vs. 400, *p* = 0.66), pain score VAS (4.2 vs. 4.1, *p* = 0.34; 3.1 vs. 3.0, *p* = 0.49; 1.3 vs. 1.4, *p* = 0.36), symptom improvement (113 vs. 103, *p* = 0.36), and recurrence (9 vs. 11, *p* = 0.44) were not significantly different; however, for the operative time (suture time 22 vs. 41, *p* = 0.00; total time 72 vs. 96, *p* = 0.01), median blood loss (45 vs. 75, *p* = 0.01), anal exhaust time (12 vs. 18, *p* = 0.00), hospital stay (2 vs. 3, *p* = 0.02), complications (3 vs. 12, *p* = 0.01), and total cost (51,231 vs. 26,899, *p* = 0.00), the difference was statistically significant. * There is no robot docking time in laparoscopic surgery, which is recorded as 0. Complications included fever, abdominal distension, bleeding, and poor incision healing. No serious complications occurred in either group, and no case was converted to laparotomy. VAS: Visual Analogue ScaleScore. * *p* < 0.05, ** *p* < 0.01.

**Table 3:** Details of hospitalization expenses.

	Robot (*n* = 125)	Laparoscope (*n* = 110)	*p* Value
Operating expenses (RMB)	31,561 (30,104–33,291)	6694 (3420–3990)	0.00 **
Other expenses (RMB)	19,870 (14,478–23,664)	20,205 (14,792–27,036)	0.66
Composition ratio of other expenses			
Consumable	39%	43%	0.78
Inspection and laboratory	27%	22%	0.65
Drug	16%	17%	0.81
Treatment	8.4%	8.7%	0.63
Nursing	6.1%	6.5%	0.45
Other	3.3%	3.2%	0.51

** *p* < 0.01.

## Data Availability

The data that support. The findings of this study are available from the corresponding author upon reasonable request.
